# Supplementary material for: Methylobacterium, a major component of the culturable bacterial endophyte community of wild Brassica seed
Source: PeerJ. 2020 Jul 10;8:e9514. doi: 10.7717/peerj.9514 (PMC7357558; doi:10.7717/peerj.9514)
Supplement: Supplemental Information 1 [file peerj-08-9514-s001.docx]

**Table S1.** Wild and landrace *Brassica* accessions screened for the presence of endophytic bacteria and fungi. Seed was stored in in the Margot Forde Germplasm Centre (MFGC) prior to this survey.

| **MFGC accession number** | **Country of origin** | **Species** | **Harvest date (seed age)** | **Altitude (m)** |
| --- | --- | --- | --- | --- |
| O2329 | unknown | *B. balearica* | unknown | unknown |
| O2330 | unknown | *B. barrelieri* | unknown | unknown |
| O2331 | Portugal | *B. barrelieri* | unknown | 888 |
| O2332 | Italy | *B. incana* | unknown | 74 |
| O2333 | unknown | *B. incana* | unknown | 13 |
| O2334 | Slovakia | *B. juncea* | unknown | 144 |
| O2335 | unknown | *B. juncea* | unknown | unknown |
| O2336 | Poland | *B. juncea* | unknown | 134 |
| O2337 | India | *B. juncea* | unknown | 248 |
| O2338 | Thailand | *B. juncea* | unknown | 93 |
| O2350 | Italy | *B. nigra* | unknown | 234 |
| O2351 | Bulgaria | *B. nigra* | unknown | 121 |
| O2352 | Germany | *B. oleracea* | unknown | 202 |
| O2353 | Germany | *B. oleracea* | unknown | 76 |
| O2354 | Spain | *B. oleracea* | unknown | 594 |
| O2355 | China | *B. rapa* | unknown | 1857 |
| O2356 | Russia | *B. rapa* | unknown | unknown |
| O2357 | Slovakia | *B. rapa* | unknown | 1843 |
| O2358 | Georgia | *Brassica sp.* | unknown | 1450 |
| O2359 | Spain | *B. barrelieri* | 1/01/1995 | 2069 |
| O2360 | Spain | *B. barrelieri* | 1/01/1996 | 27 |
| O2361 | Turkey | *B. deflexa* | 1/01/1995 | 940 |
| O2362 | Morocco | *B. densnottesii* | 1/01/1996 | 2015 |
| O2363 | Iran | *B. elongata* | 1/01/1995 | 1301 |
| O2364 | Spain | *B. fruticulosa* | 1/01/1997 | 206 |
| O2365 | Algeria | *B. fruticulosa* | 1/01/1994 | 306 |
| O2366 | Algeria | *B. fruticulosa* | 1/01/1994 | 140 |
| O2367 | Algeria | *B. gravinae* | 1/01/1994 | 1749 |
| O2368 | India | *B. juncea* | 1/01/1999 | 251 |
| O2369 | Zambia | *B. juncea* | 1/01/1994 | 1090 |
| O2370 | Zambia | *B. juncea* | 1/01/1994 | 1257 |
| O2371 | Germany | *B. juncea* | 1/01/1997 | 202 |
| O2372 | Mongolia | *B. juncea* | 1/01/2000 | 1335 |
| O2373 | Spain | *B. nigra* | 1/01/1994 | 19 |
| O2374 | Ethiopia | *B. nigra* | 1/01/1994 | 1320 |
| O2375 | Germany | *B. nigra* | 1/01/1996 | *73* |
| O2376 | Israel | *B. nigra* | 1/01/1999 | 503 |
| O2377 | USA | *B. rapa* | 1/01/2010 | 23 |
| O2378 | USA | *B. rapa* | 1/01/2010 | 23 |
| O2379 | USA | *B. rapa* | 1/01/2010 | 23 |
| O2380 | USA | *B. rapa* | 1/01/2010 | 23 |
| O2381 | USA | *B. rapa* | 1/01/2010 | 23 |
| O2382 | USA | *B. rapa* | 1/01/2010 | 15 |
| O2383 | USA | *B. rapa* | 1/01/2010 | 15 |
| O2384 | Egypt | *B. rapa* | 1/01/1996 | 3 |
| O2385 | India | *B. rapa* | 1/01/2000 | 176 |
| O2386 | Spain | *B. repanda* | 1/01/2011 | 8 |
| O2387 | Spain | *B. repanda* | 1/01/2007 | 400 |
| O2388 | India | *Brassica sp.* | 1/01/2010 | 279 |
| O2389^*^ | Finland | *B. napus* | 1/01/1983 | 158 |
| O2390^*^ | Finland | *B. napus* | 1/01/1991 | 158 |
| O2391^*^ | Sweden | *B. napus* | 1/01/2000 | 16 |
| O2392^*^ | Iceland | *B. napus* | 1/01/1988 | 733 |
| O2393^*^ | Iceland | *B. napus* | 1/01/1988 | 733 |
| O2394^*^ | Iceland | *B. napus* | 1/01/2010 | 733 |
| O2395^*^ | Iceland | *B. napus* | 1/01/1988 | 733 |
| O2396^*^ | Iceland | *B. napus* | 1/01/1988 | 733 |
| O2397^*^ | Iceland | *B. napus* | 1/01/1988 | 733 |
| O2398^*^ | Iceland | *B. napus* | 1/01/1988 | 733 |
| O2399^*^ | Iceland | *B. napus* | 1/01/1988 | 733 |
| O2400^*^ | Iceland | *B. napus* | unknown | 733 |
| O2401^*^ | Norway | *B. napus* | 1/01/1996 | 759 |
| O2402^*^ | Sweden | *B. napus* | 1/01/2000 | 16 |
| O2403^*^ | Sweden | *B. napus* | 1/01/1999 | 16 |
| NZ01 | New Zealand | *Brassica sp.* | 1/05/2014 | 28 |
| NZ02 | New Zealand | *Brassica sp.* | 1/05/2014 | 28 |
| NZ03 | New Zealand | *Brassica sp.* | 1/05/2014 | 28 |
| NZ04 | New Zealand | *Brassica sp.* | 1/05/2014 | 28 |
| NZ05 | New Zealand | *Brassica sp.* | 1/05/2014 | 28 |
| NZ06 | New Zealand | *Brassica sp.* | 1/05/2014 | 28 |
| NZ07 | New Zealand | *Brassica sp.* | 1/05/2014 | 28 |
| NZ08 | New Zealand | *Brassica sp.* | 1/05/2014 | 28 |
| NZ09 | New Zealand | *Brassica sp.* | 1/05/2014 | 28 |
| NZ10 | New Zealand | *Brassica sp.* | 1/05/2014 | 28 |
| NZ11 | New Zealand | *Brassica sp.* | 1/05/2014 | 28 |
| NZ12 | New Zealand | *Brassica sp.* | 1/05/2014 | 28 |
| NZ13 | New Zealand | *Brassica sp.* | 1/05/2014 | 28 |
| NZ14 | New Zealand | *Brassica sp.* | 1/05/2014 | 28 |
| NZ15 | New Zealand | *Brassica sp.* | 1/05/2014 | 28 |
| NZ16 | New Zealand | *Brassica sp.* | 1/05/2014 | 28 |
| NZ17 | New Zealand | *Brassica sp.* | 1/05/2014 | 28 |
| NZ18 | New Zealand | *Brassica sp.* | 1/05/2014 | 28 |
| NZ19 | New Zealand | *Brassica sp.* | 1/05/2014 | 28 |

*Landrace
