# Supplementary figures and images for: Methylobacterium, a major component of the culturable bacterial endophyte community of wild Brassica seed
Source: PeerJ. 2020 Jul 10;8:e9514. doi: 10.7717/peerj.9514 (PMC7357558; doi:10.7717/peerj.9514)

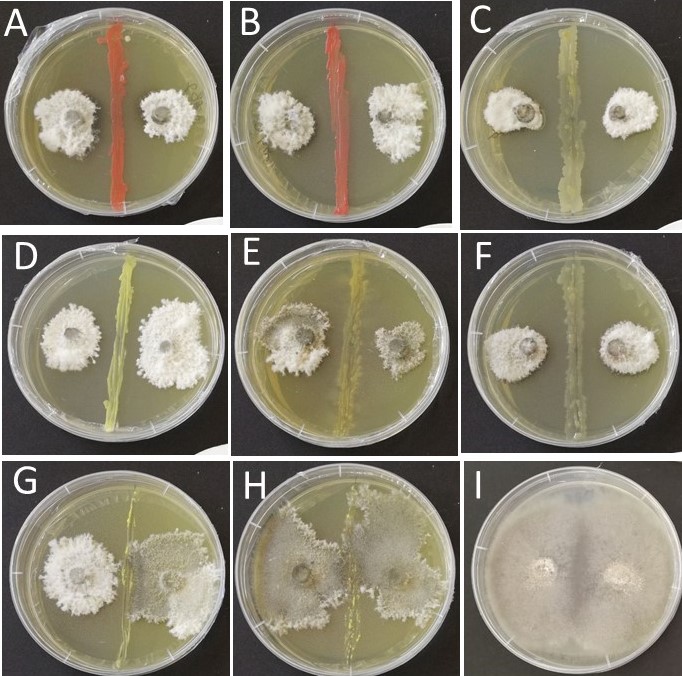

Supplement: Supplemental Information 2 — (A) Methylobacterium fujisawaense B82. (B) Methylobacterium phyllosphaerae B64. (C) Novosphingobium resinovorum B26. (D) Plantibacter flavus B47. (E) Stenotrophomonas rhizophila B3. (F) Pseudomonas lactis B35. (G) Sphingomonas insulae B111. (H) Sphingomonas yantingensis B31. (I) pathogen-only control plate inoculated with L. maculans. [file peerj-08-9514-s002.jpg]
